# Supplementary figures and images for: Prediction of the development of delirium after transcatheter aortic valve implantation using preoperative brain perfusion SPECT
Source: PLoS One. 2022 Nov 3;17(11):e0276447. doi: 10.1371/journal.pone.0276447 (PMC9632803; doi:10.1371/journal.pone.0276447)

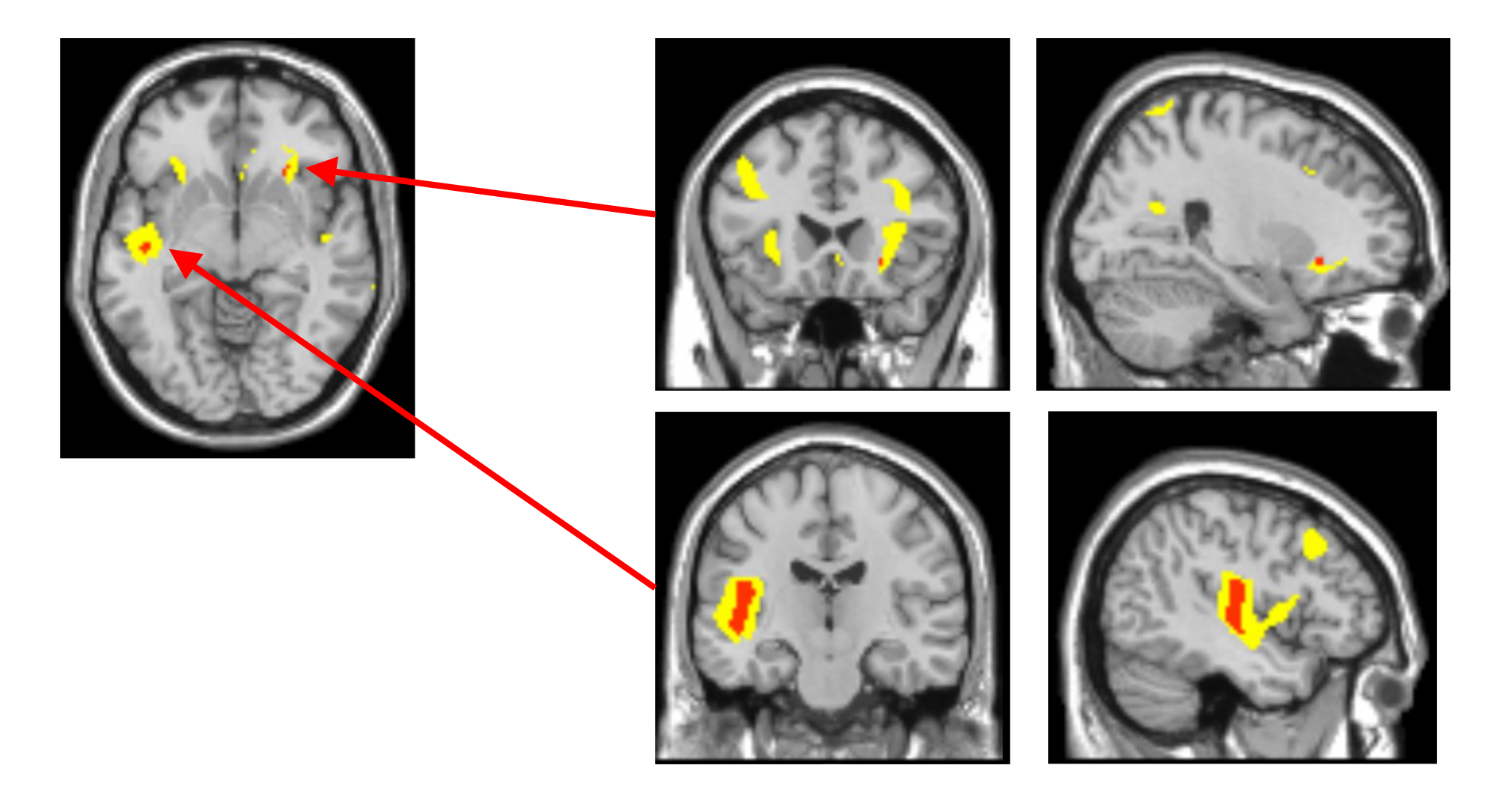

Supplement: S1 Fig — Results of the whole brain voxel-wise analysis at a significance threshold of P<0.05 with family-wise error corrections (red regions) and P<0.001 without multiple comparisons (yellow regions). (TIF) [file pone.0276447.s001.tif]
