# Supplementary material for: Prediction of the development of delirium after transcatheter aortic valve implantation using preoperative brain perfusion SPECT
Source: PLoS One. 2022 Nov 3;17(11):e0276447. doi: 10.1371/journal.pone.0276447 (PMC9632803; doi:10.1371/journal.pone.0276447)
Supplement: Attachment — Submitted filename: Responses to Reviewer.docx [file pone.0276447.s002.docx]

**Responses to Reviewer 1**

**Manuscript: PON-D-22-15512**

**Authors: Takeuchi M, et al.**

**Title: Prediction of the development of delirium after transcatheter aortic valve implantation using preoperative brain perfusion SPECT**

**In this manuscript by Takeuchi et al., “Prediction of the development of delirium after transcatheter aortic valve implantation using preoperative brain perfusion SPECT” was shown. This is very excellent study.**

(Response)

We are grateful for receiving the favorable comment from the Reviewer. Please see the responses to the Reviewer’s comments below, which we hope improve the revised manuscript substantially.

**#1: Hypoactive delirium**

**Most of the DAT might be hypoactive delirium. The type of the delirium should be explained more specifically through the manuscript.**

(Response)

We agree that most of the DAT was hypoactive. As shown in Table 3 below, hypoactive delirium was the most common type of delirium, accounting for 66.6% of the cases. A previous study also reports a consistent finding (Meagher D, et al. *J Neuropsychiatry Clin Neurosci****.*** 2008;20:185–193).

|  | DAT group | Non-DAT group | P value |
| --- | --- | --- | --- |
| N | 12 (24%) | 38 (76%) | － |
| Type of delirium |  |  |  |
| **Hypoactive** | **8 (66.6)** | **NA** | **－** |
| Hyperactive | 2 (16.7) | NA | － |
| Mixed | 2 (16.7) | NA | － |
| Duration, day | 2.67±1.84 | NA | － |

As the original study design in this study, however, we performed the neuroimaging analyses without the classification of the three types of delirium (Fig 2). To specify associations of hypoactive delirium with cerebral perfusion, we have compared CBF of patients with hypoactive delirium (N=8) with that of non-DAT group. The results were similar to those comparing DAT group with non-DAT group: CBF in the bilateral insula was lower in patients with hypoactive delirium than in non-DAT group (P<0.05 FWE corrected) (S1 Fig). DE% of the bilateral insula in patients with hypoactive delirium was greater than the non-DAT group (16.6±9,8% vs. 7.0±11.3%, P=0.03).

To reflect these comments, we have added the contents as follows:

(sentences 193–198)

To specify associations of hypoactive delirium, the most common type of DAT, with cerebral perfusion, we have also compared CBF of patients with hypoactive delirium with that of non-DAT group. The results were similar to those comparing DAT group with non-DAT group: CBF in the insula was lower in patients with hypoactive delirium than in non-DAT group (P<0.05 FWE corrected) (S1 Fig). DE% of the insula in patients with hypoactive delirium was greater than the non-DAT group (16.6±9,8% vs. 7.0±11.3%, P=0.03).

(sentences 223–224)

CBF in the insula was lower also in patients with hypoactive delirium than the non-DAT group.

(sentences 286–289)

**S1 Fig. Lower insular perfusion in patients with hypoactive delirium compared with the non-DAT group.** Results of the whole brain voxel-wise analysis at a significance threshold of P<0.05 with family-wise error corrections (red regions) and P<0.001 without multiple comparisons (yellow regions).

**#2: CBF in the right insular cortex**

**Reduced CBF in the right insular cortex seems to be associated with DAT. Please specify this through the manuscript. Especially, in the Table 4, the relationship between CBF in the right insular cortex and the delirium was not clear.**

(Response)

We agree that our data are not enough to decide whether only the right or left insula is associated with DAT and have reduced the emphasis on the laterality of the association of insular CBF with DAT in the revised manuscript. The laterality of brain abnormalities associated with DAT should be investigated in the future studies.

We have thus specified the lower regional CBF in the insula in the DAT group relative to the non-DAT group through the manuscript as below. Whole brain voxel-wise analyses were re-tested at both exploratory threshold of P<0.001 uncorrected for multiple comparisons and more stringent threshold of P=0.05 with family-wise error (FWE) corrections for multiple comparisons (Fig 2 and S1 Fig [please see our response to the Reviewer’s comment #1]). Logistic regression analyses in Fig 3 and Table 4 have been re-perforemd using DE% of the bilateral insula.

(sentences 45–51)

The whole brain analysis showed that preoperative CBF in the insula was lower in the DAT than in the non-DAT group (P<0.05, family-wise error correction). Decrease extent ratio in the insula of the DAT group (17.6±11.5%) was also greater relative to that of the non-DAT group (7.0±11.3%) in the region of interest-based analysis (P=0.007). A model that included preoperative CBF in the insula and conventional indicators (frailty index, short physical performance battery and mini-mental state examination) showed the best predictive power for DAT (AUC 0.882).

(sentences 54,55)

These results suggest that preoperative CBF in the insula is associated with DAT and may be useful for its prediction.

(sentences 146–148)

Results of the voxel-wise analysis were expressed at both exploratory threshold of P<0.001 uncorrected for multiple comparisons and more stringent threshold of P=0.05 with family-wise error (FWE) corrections for multiple comparisons.

(sentences 187–191)

The whole-brain voxel-wise analysis showed that preoperative regional CBF in the insular cortex was lower in the DAT group than in the non-DAT group (P>0.05 FWE corrected) (Fig 2A–C). This was supported by the ROI-based analysis; DE% of the insula was significantly greater in the DAT group than in the non-DAT group (17.6±11.5% vs. 7.0±11.3%, P=0.007) (Fig 2D).

(sentences 203–207)

Of these, CBF in the insula had the highest AUC (0.818). An addition of CBF in the insula increased AUC of the model predicting DAT with each of frailty index, MMSE and SPPB (Fig 3). Although not achieving a significant difference from other models (P>0.05), the model including all of frailty index, MMSE, SPPB and CBF in the insula had the highest AUC (0.882) (Fig 3).

(sentences 214–216)

The addition of insular CBF to these traditional preoperative risk factors increased an accuracy of the model predicting DAT with frailty index, MMSE and SPPB (AUC 0.882).

(sentences 523–529)

**Fig 2. Lower insular perfusion in the DAT group compared with the non-DAT group.**

Results of the whole brain voxel-wise analysis shown in the sagittal (A), axial (B), and the coronal slices (C) (red areas, P<0.05 with family-wise error corrections; yellow areas, P<0.001 without corrections for multiple comparisons). In the region of interest-based analysis, decrease extent ratios were higher in the DAT group (N=12) than in the non-DAT group (N=38) (D). Abbreviations: DAT, delirium after transcatheter aortic valve implantation.

(sentences 522–527)

**Fig 3. Results of multivariable logistic regression analyses with an addition of CBF in the insula to classical indicators.**

Abbreviations: AUC, area under curve; CBF, cerebral blood flow; Ins, insular;

MMSE, mini mental state examination; SPPB, short physical performance battery.

(sentences 557–560)

**Table 4. Results of univariable regression analyses with delirium as an outcome.**

|  | Odds ratio | 95% confidence interval | P value | Area under curve |
| --- | --- | --- | --- | --- |
| Short physical performance battery | 0.855 | (0.711, 1.028) | 0.096 | 0.701 |
| Mini mental state examination | 0.757 | (0.625, 0.916) | 0.004 | 0.787 |
| Frail index | 2.037 | (1.168, 3.552) | 0.012 | 0.734 |
| Cerebral blood flow in the insula | 1.071 | (1.011, 1.135) | 0.021 | 0.818 |

Only the results with P<0.10 are shown. Cerebral blood flow in the insula was expressed as decrease extent ratio from the region of interest-based analysis.

**#3: Diuretic use**

**Please provide the information for the diuretic use, which might be associated with the study results.**

(Response)

We have added information on diuretics to Table 2. There was no difference in diuretic use between the two groups.

**Table 2. Preoperative drug use.**

|  | DAT group | Non-DAT group | P value |
| --- | --- | --- | --- |
| N | 12 (24%) | 38 (76%) | － |
| sleep medication, anti-anxiety drug | 3 (25.0) | 18 (47.4%) | 0.240 |
| Antidepressant | 3 (25.0) | 2 (5.3) | 0.082 |
| atypical psychotropic drug | 2 (16.7) | 1 (2.6) | 0.139 |
| Steroid | 1 (8.3) | 4 (10.5) | 1.000 |
| Opioid | 1 (8.3) | 1 (2.6) | 0.426 |
| NSAIDs | 5 (41.7) | 11 (29.0) | 0.186 |
| Benzodiazepines | 3 (25.0) | 15 (39.5) | 0.497 |
| Digitalis | 1 (8.3) | 0 (0.0) | 0.240 |
| β-blocker | 6 (50.0) | 15 (39.5) | 0.738 |
| antiarrhythmic drug | 2 (16.7) | 12 (31.6) | 0.468 |
| oral cardiotonic drug | 0 (0.0) | 2 (5.3) | 1.000 |
| Diuretic | 3 (25.0) | 11 (29.0) | 1.000 |

**#4: Low EF/low gradient**

**In this study population, the EF seems to be preserved. Might be there some patients with AS with low EF and/or low gradient? Some patients were assessed severe AS using dobutamine?**

**#5: Diastolic function**

**Diastolic function might be associated with delirium. Please provide the information such as the E/e’.**

(Response)

As you pointed out, majority of the participants (82%) had preserved LVEF (≥50%). This is at least partly because emergent cases and patients with NYHA functional class IV were excluded in this study. As shown in Table 1, all of the 3 patients with low-flow and low gradient AS who required the assessment using dobutamine were included in the non-DAT group.

We have also added E/e' as an indicator of diastolic function to Table 1. No difference in E/e' was found between the two groups.

|  | DAT group | Non-DAT group | P value |
| --- | --- | --- | --- |
| N | 12 (24%) | 38 (76%) | － |
| Echocardiography |  |  |  |
| Left ventricular ejection fraction, % | 64.0±6.4 | 61.4±14.0 | 0.377 |
| E/e’ | 22.8±10.9 | 19.6±9.2 | 0.404 |
| Aortic valve area, cm^2^ | 0.61±0.12 | 0.69±0.26 | 0.180 |
| Peak velocity, m/s | 4.62±0.95 | 4.30±0.94 | 0.310 |
| Aortic mean gradient, mmHg | 51.8±21.2 | 45.5±21.1 | 0.379 |
| Low flow, Low gradient AS | 0 (0.0) | 3 (7.9) | 1.000 |

**Table 1. Baseline patient characteristics.**

**#6: Regional cerebral blood flow**

**It would be helpful if the specific description for CBF was provided. In the Fig3 and Fig4, CBF sounds like global CBF. Thus, use of the term such as “the reduced CBF in the right insular cortex” would be helpful.**

(Response)

Our apologies for the inaccurate expressions. We have specified the descriptions of regional CBF in Fig 3 (please see our response to the Reviewer’s comment #2) and Fig 4 as below.

(sentences 536–540)

**Fig 4. Multifactorial nature of DAT and possible involvement of preoperative CBF in the insula.**

Significant (P>0.10) factors that contribute the onset of DAT in the present study are emphasized as red-front. Abbreviations: CBF, cerebral blood flow; DAT, delirium after transcatheter aortic valve implantation.

**#7: Discussion for the relationship between reduced CBF in the right insular cortex and delirium**

**It would be clear if the relationship between reduced CBF in the right insular cortex and delirium was discussed more specifically. The main symptoms were anergia or hypoactivity like depressive symptom?**

(Response)

As discussed in our response to the Reviewer’s comment #2, we have reduced the emphasis on the laterality of the association of insular CBF with DAT in the revised manuscript. For this purpose, we have added discussions as below:

(sentences 238–245)

The left insular is associated with activity of the parasympathetic nervous system, while sympathetic arousal occurs in stimulation of the right insular [38,41]. Patients with right insular lesions show delirium-like behaviors compared with those with non-insular or left insular lesions [40]. In the present study, however, CBF in the bilateral insula was lower in patients with hypoactive delirium compared to the non-DAT group. The DAT group, including all of the three types of delirium, also showed a similar tendency of lower CBF in the bilateral insula than the non-DAT group. The laterality of brain abnormalities associated with DAT should be investigated in the future studies.

**#8: Symptom change**

**It is interesting if the change of the symptom such as chest pain was provided. In the delirium patients, the chest pain**

(Response)

In this study population, chest pain appeared in some patients before TAVI while, fortunately, no patient in both DAT and non-DAT groups complained it after TAVI.

**#9: Stroke during TAVI procedure**

**Was there any patients with stroke during TAVI procedure?**

(Response)

As shown in Fig 1 below, complications after TAVI were excluded and no cerebrovascular events occurred during the procedure.
